# High procalcitonin levels associated with increased intensive care unit admission and mortality in patients with a COVID-19 infection in the emergency department

**DOI:** 10.1186/s12879-022-07144-5

**Published:** 2022-02-21

**Authors:** Kirby Tong-Minh, Yuri van der Does, Susanna Engelen, Evelien de Jong, Christian Ramakers, Diederik Gommers, Eric van Gorp, Henrik Endeman

**Affiliations:** 1 Department of Emergency Medicine, Erasmus University Medical Center, Nc-017k, Postbus 2040, 3000 CA Rotterdam, The Netherlands; 2 Department of Intensive Care, Rode Kruis Ziekenhuis, Beverwijk, The Netherlands; 3 Department of Clinical Chemistry, Erasmus University Medical Center, Rotterdam, The Netherlands; 4 Department of Intensive Care, Erasmus University Medical Center, Rotterdam, The Netherlands; 5 Department of Internal Medicine, Erasmus University Medical Center, Rotterdam, The Netherlands; 6 Department of Viroscience, Erasmus University Medical Center, Rotterdam, The Netherlands

**Keywords:** COVID-19, Biomarkers, Emergency department, Procalcitonin

## Abstract

**Background:**

Patients with a severe COVID-19 infection often require admission at an intensive care unit (ICU) when they develop acute respiratory distress syndrome (ARDS). Hyperinflammation plays an important role in the development of ARDS in COVID-19. Procalcitonin (PCT) is a biomarker which may be a predictor of hyperinflammation. When patients with COVID-19 are in the emergency department (ED), elevated PCT levels could be associated with severe COVID-19 infections. The goal of this study is to investigate the association between PCT levels and severe COVID-19 infections in the ED.

**Methods:**

This was a retrospective cohort study including patients with a confirmed COVID-19 infection who visited the ED of Erasmus Medical Center in Rotterdam, the Netherlands, between March and December 2020. The primary outcome was a severe COVID-19 infection, which was defined as patients who required ICU admission, all cause in-hospital mortality and mortality within 30 days after hospital discharge. PCT levels were measured during the ED visit. We used logistic regression to calculate the odds ratio (OR) with 95% confidence interval (95% CI) and corresponding area under the curve (AUC) of PCT on a severe COVID-19 infection, adjusting for bacterial coinfections, age, sex, comorbidities, C-reactive protein (CRP) and D-dimer.

**Results:**

A total of 332 patients were included in the final analysis of this study, of which 105 patients reached the composite outcome of a severe COVID-19 infection. PCT showed an unadjusted OR of 4.19 (95%CI: 2.52–7.69) on a severe COVID-19 infection with an AUC of 0.82 (95% CI: 0.76–0.87). Corrected for bacterial coinfection, the OR of PCT was 4.05 (95% CI: 2.45–7.41). Adjusted for sex, bacterial coinfection, age any comorbidity, CRP and D-dimer, elevated PCT levels were still significantly associated with a severe COVID-19 infection with an adjusted OR of 2.11 (95% CI: 1.36–3.61). The AUC of this multivariable model was 0.85 (95%CI: 0.81–0.90).

**Conclusion:**

High PCT levels are associated with high rates of severe COVID-19 infections in patients with a COVID-19 infection in the ED. The routine measurement of PCT in patients with a COVID-19 infection in the ED may assist physicians in the clinical decision making process regarding ICU disposition.

**Supplementary Information:**

The online version contains supplementary material available at 10.1186/s12879-022-07144-5.

## Background

Coronavirus disease (COVID-19) caused by the novel Coronavirus (SARS-CoV-2) was declared a pandemic on the 11th of March 2020 by the World Health Organization [1]. Ever since, COVID-19 caused a high burden on hospital and intensive care unit capacity [2]. Patients with COVID-19 often require hospitalization and treatment at an intensive care unit (ICU) when they develop acute respiratory distress syndrome (ARDS). The exact underlying pathophysiology of ARDS in these severe COVID-19 infections is under research with an exponentially growing amount of studies on this topic [3, 4]. Many studies have found that hyperinflammation, caused by an overwhelming upregulation of the immune system, and immune thrombosis play a major role in the development of ARDS in COVID-19 [5].

When patients with a COVID-19 infection present at the emergency department (ED), it is important to be able to identify patients who are at high risk of developing a severe COVID-19 infection. These patients may benefit from more extensive monitoring or early ICU admission.

Different biomarkers have been identified as predictors of disease severity in COVID-19 [6], including procalcitonin (PCT). PCT is a biomarker previously used for distinguishing viral infections from bacterial infections [7], although its use in the ED for this purpose is controversial [8, 9]. Elevated PCT levels are often seen in patients with a bacterial infection. PCT is the prohormone of calcitonin and in a physiological state produced by C-cells of the thyroid gland. During inflammation, PCT is synthesized in all tissues. Bacterial toxins are well known triggers of synthesis of PCT. Other triggers of synthesis include interleukin-6 and tumor necrosis factor alpha (TNF-alpha) [10, 11]. High levels of these cytokines have been reported in severe COVID-19 infections [12]. For this reason, PCT may also be elevated in a hyperinflammatory state in the absence of a bacterial pathogen. ARDS is clinically the most important severe complication of COVID-19, therefore PCT may be predictive of hyperinflammation and the development of ARDS [13]. This may aid physicians in the ED to identify patients that are at risk of developing ARDS early.

The goal of this study is to investigate the association between PCT levels and severe COVID-19 infections in the ED.

## Methods

In this retrospective single center cohort study we included patients with a confirmed COVID-19 infection who visited the ED of Erasmus University Medical Center, in Rotterdam, the Netherlands, between 1 March 2020 and 31 December 2020. Erasmus University Medical Center is an academic hospital with 40.000 ED visits annually. This study was conducted in accordance with the Declaration of Helsinki (64th WMA General Assembly, Fortaleza, Brazil, October 2013). The institutional review board waivered informed consent for the retrospective use of clinical data of COVID-19 patients. The study was performed following the STROBE guidelines (Additional file 1).

### Inclusion and exclusion criteria

Patients were included if they tested positive on COVID-19 by nasal pharyngeal PCR test on the day of the ED visit, the reason for ED visit was related to the COVID-19 infection and PCT levels were measured in the ED. Patients were excluded if the PCT levels were measured but invalid (e.g. hemolytic blood samples), if the COVID-19 infection was a secondary finding and not the primary reason for ED visit or when no follow-up data were available due to transfer to another hospital.

### Data collection

Patient data including demographics, comorbidities, vital signs and laboratory tests were collected from the electronic patient records. Vital signs including heart rate, arterial oxygen saturation, respiratory rate, temperature and blood pressure were recorded from the time of ED visit. Laboratory testing during the ED visit included hemoglobin, red cell distribution width (RDW), leucocyte count, thrombocytes, total bilirubin, alanine aminotransferase (ALAT), aspartate aminotransferase (ALAT), lactate, D-dimer, C-reactive protein (CRP) and PCT. Patients were followed up for 30 days after hospital discharge. Patient disposition from the ED was categorized into discharge home from the ED, admission to general ward and admission to the ICU. Mortality data was classified as all cause in-hospital mortality and mortality at home within 30 days after hospital discharge. To correct for potential bacterial coinfections at ED visit, culture within 48 h of ED visit were reviewed. If blood, urine or sputum cultures or pneumococcal or legionella antigen tests were positive, patients were classified as having a bacterial coinfection.

### Primary outcome

The primary outcome was a severe COVID-19 infection, defined as patients that were admitted to the ICU or patients that died by any in-hospital or within 30 days after hospital discharge. We calculated odds ratios (ORs) for PCT as continuous variable and commonly used cut-off values of PCT of 0.25 ng/mL, 0.5 ng/mL and 1.0 ng/mL. These cut-off values are derived from recommendations in previous literature [14].

### Secondary outcomes

The secondary outcomes of this study were hospital admission, ICU admission and all-cause mortality, either in-hospital or within 30 days after hospital discharge. For the secondary outcomes, PCT was analyzed as both continuous variable and at the different cut-off values as previously described.

### PCT measurement

PCT analysis was available as a standard laboratory test in patients who visited the ED with a suspected COVID-19 infection. Blood was collected in a lithium heparin tube and analyzed directly upon arrival in the clinical chemistry laboratory. PCT was measured using E801 Elecsys BRAHMS PCT reagent on a COBAS 8000 (Roche Diagnostics, Switzerland). The PCT values were available to the treating physician during the ED visit.

### Statistical analysis

Normally distributed variables were reported as mean with standard deviation (SD), non-normally distributed variables as median with interquartile range (IQR). Multiple imputation was used for handling missing data.

Differences in dichotomous variables between the severe and non-severe COVID-19 infection patients were analyzed with a chi-square test. Differences in continuous variables were analyzed using an independent sample T-test for normally distributed data and a Mann–Whitney-U test for non-normally distributed data. Following, we did an univariate logistic regression analysis calculating the ORs of the variables that significantly differed between the groups.

For the primary outcome, we calculated unadjusted odds ratios of PCT using univariate logistic regression analysis. We calculated adjusted ORs of PCT correcting for only bacterial coinfections and in a multivariable logistic regression model correcting for the following predefined variables: age, sex, bacterial coinfection, the presence of any comorbidity, CRP and D-dimer. We calculated receiver operating characteristic (ROC) curves and the corresponding area under the curve (AUC). The calibration statistics of the univariate analysis of PCT and the multivariable regression model were analyzed using the Hosmer-Lemshow test to investigate the goodness-of-fit and graphically reported by calibration plot with intercept and slope statistics.

For the secondary outcomes, we used predefined cut-off values of 0.25 ng/mL, 0.5 ng/mL, 1.0 ng/mL of PCT and calculated the unadjusted ORs, the sensitivity, specificity, negative predictive value and positive predictive value on the primary and secondary outcomes of the study.

The correct for the skewed distribution of PCT, we transformed PCT using the natural logarithm and performed the univariate analysis and calibration analysis in Additional file 3. Additionally, we transformed PCT into a nominal variable with the ranges of 0–0.25 ng/mL (reference), 0.25–0.50 ng/mL, 0.50–1.00 ng/mL and larger than 1.00 ng/mL and used this in the multivariable logistic regression model in Additional file 4.

Statistical analyses were performed using ‘R’ version 4.1.1. We used the MICE package for multiple imputation of missing data and the RMS package for calibration statistics.

## Results

**Fig. 1:**
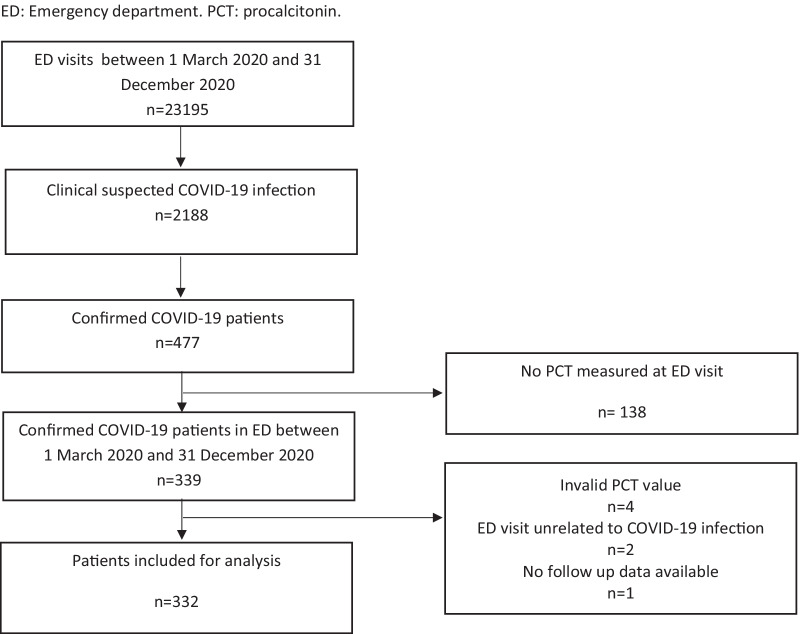
Flow diagram of included patients. *ED* emergency department, *PCT* procalcitonin

Between 1 March 2020 and 31 December 2020, a total of 23,195 patients visited the ED of which 2188 were clinically suspected of having a COVID-19 infection. Of these patients, 477 patients had a positive COVID-19 PCR-test at the day of the ED visit. Procalcitonin was measured in 339 of these patients. Four patients were excluded due to an invalid procalcitonin measurement, two patients were excluded because the ED visit was not related to the COVID-19 infection and one patient was excluded because there was no follow up data available. A total of 332 patients were included in the analysis of this study (Fig. 1). There were missing data in 3% of the data These missing data were imputated using multiple imputation. A specification of missing data per variable is shown in Additional file 2.

A total of 105 (32%) patients reached the composite outcome of a severe COVID-19 infection. Of these 105 patients, 44 (13%) were admitted to the ICU and 61 (18%) patients died. The non-severe COVID-19 infection group consisted of 227 (68%) patients, of which 67 (20%) were discharged directly from the ED and 158 (48%) were discharged from the general ward after an uncomplicated hospital admission. Baseline characteristics and differences between both groups including unadjusted ORs are shown in Table 1. The severe COVID-19 group was significantly older, more often male and had more frequently diabetes mellitus and any form of immunodeficiency as comorbidity. In vital parameters there were significant differences in heartrate, respiratory rate, oxygen saturation and diastolic blood pressure. In laboratory testing there were significant differences in PCT, CRP, leukocyte count, RDW, ASAT, ALAT, lactate, D-dimer and creatinine. Patients with severe COVID-19 infections had a bacterial coinfection more often.

**Table 1:** Baseline characteristics and univariate analysis

Patient characteristics		All patients	Non-severe COVID-19 infection	Severe COVID-19 infection	p-value	Unadjusted OR (95% CI)
Demograpic data			n = 227	n = 105		
Sex: male	n (%)	191 (57.5)	115 (50.7)	76 (72.4)	< 0.001	2.55 (1.56—4.26)
Age	Mean (SD)	60 (16.4)	58.1 (16.1)	64.9 (16.3)	< 0.001	1.03 (1.01–1.05)
Comorbidity: pulmonary disease	n (%)	83 (25)	53 (23.8)	29 (27.6)	0.54	
Comorbidity: cardiovascular disease	n (%)	165 (49.7)	115 (50.7)	50 (47.6)	0.691	
Comorbidity: diabetes mellitus	n (%)	89 (26.8)	49 (21.6)	40 (38.1)	0.002	2.23 (1.35—3.71)
Comorbidity: malignancy	n (%)	52 (15.7)	35 (15.4)	17 (16.1)	0.986	
Comorbidity: renal disease	n (%)	55 (16.6)	43 (18.9)	12 (11.4)	0.12	
Comorbidity: auto-immune diseases	n (%)	37 (11.1)	27 (11.9)	10 (9.5)	0.652	
Comorbidity: immunodeficiency	n (%)	63 (19)	53 (23.3)	10 (9.5)	0.005	
*Vital parameters*						
Heartrate	Mean (SD)	94.1 (18.4)	91 (17.9)	101 (19.6)	< 0.001	1.03 (1.02–1.04)
Respiratory rate	Mean (SD)	25.2 (9)	24 (8.9)	27 (8.9)	0.001	1.03 (1.02–1.07)
Oxygen saturation	Median (IQR)	95 (94–97)	96 (94–97)	94 (88–96)	< 0.001	0.85 (0.82–0.92)
Diastolic blood pressure	Mean (SD)	79.4 (16)	81 (15.6)	77 (16.2)	0.005	0.98 (0.96–0.99)
Systolic blood pressure	Mean (SD)	134.6 (22.2)	136 (21.8)	135 (23.2)	0.156	
Temperature (Celcius)	Mean (SD)	37.6 (1)	37.5 (0.9)	37.8 (1.2)	0.288	
*Laboratory testing*						
Procalcitonin (ng/mL)	Median (IQR)	0.14 (0.07–0.38)	0.1 (0.06–0.18)	0.47 (0.17–1.46)	< 0.001	4.19 (2.52–7.69)
CRP (mg/L)	Median (IQR)	62 (26–136)	46 (19–93)	141 (67–213)	< 0.001	1.01 (1.01–1.02)
Leucocyte count	Median (IQR)	6.4 (4.8–9)	5.8 (4.4–7.7)	7.7 (6.1–11.1)	< 0.001	1.16 (1.1–1.24)
Hemoglobin (mmol/L)	Mean (SD)	8 (1.3)	8 (1.2)	7.9 (1.5)	0.504	
RDW (%)	Median (IQR)	13.3 (12.6–14.6)	13.2 (12.5–14.2)	13.9 (12.9–14.6)	0.014	1.04 (0.95–1.15)
Thrombocytes	Median (IQR)	206 (164–265)	205 (167–264)	206 (160–266)	0.851	
ASAT (U/L)	Median (IQR)	41 (28–61)	36 (27–53)	53 (36–81)	< 0.001	1.02 (1.01–1.02)
ALAT (U/L)	Median (IQR)	28 (19–48)	27 (18–43)	31 (22–53)	0.012	1.01 (1–1.01)
Total bilirubin (umol/L)	Median (IQR)	8 (6–11)	8 (6–11)	8 (6–12)	0.976	
D-dimer (mg/L)	Median (IQR)	1.07 (0.5–2.18)	1 (0.47–1.48)	1.72 (0.71–4.51)	< 0.001	1.22 (1.13–1.4)
Creatinin (umol/L)	Median (IQR)	88 (70–122)	82 (67–110)	111 (83–163)	< 0.001	1.01 (1–1.01)
Lactate (mmol/L)	Median (IQR)	1.3 (1–1.8)	1.2 (1–1.7)	1.6 (1.2–2.5)	< 0.001	2.15 (1.56–2.94)
*Bacterial coinfection*	n (%)	37 (11)	19 (8)	18 (17)	0.030	2.26 (1.13–4.54)
*Outcome data*						
Admission length	Median (IQR)	7 (214)	6 (09)	16 (827)	< 0.001	
Mortality	n (%)	61 (18.4)	0 (0)	66 (58.1)	< 0.001	

*SD* standard deviation, *IQR* interquartile range, *OR* odds ratio, *CRP* C-reactive protein, *RDW* red blood cell distribution width, *ASAT* aspartate aminotransferase, *ALAT* alanine-aminotransferase

PCT showed an unadjusted OR of 4.19 (95%CI: 2.52–7.69) on a severe COVID-19 infection with an AUC of 0.82 (CI: 0.76–0.87). Corrected for bacterial coinfection, high PCT remained associated with an increase in severe COVID-19 infections with an adjusted OR of 4.05 (95% CI: 2.45–7.41). In the multivariable model adjusted for sex, bacterial coinfection, age, any comorbidity, CRP and d-dimer, PCT was still significantly associated with a severe COVID-19 infection with an adjusted OR of 2.11 (95% CI: 1.36–3.61) (Table 2). The AUC of the multivariable model was 0.85 (95% CI: 0.81–0.90).

**Table 2:** Multivariable logistic regression model

Predictor	Odds ratio	Confidence interval
Procalcitonin (ng/mL)	2.11	1.36–3.61
Bacterial coinfection	1.95	0.81–4.61
Sex: male	1.32	0.72–2.44
Age	1.03	1.01–1.05
Comorbidity: any	0.97	0.44–2.19
CRP	1.01	1.00–1.01
D-dimer	1.24	1.12–1.41

*CRP* C-reactive protein

**Fig. 2:**
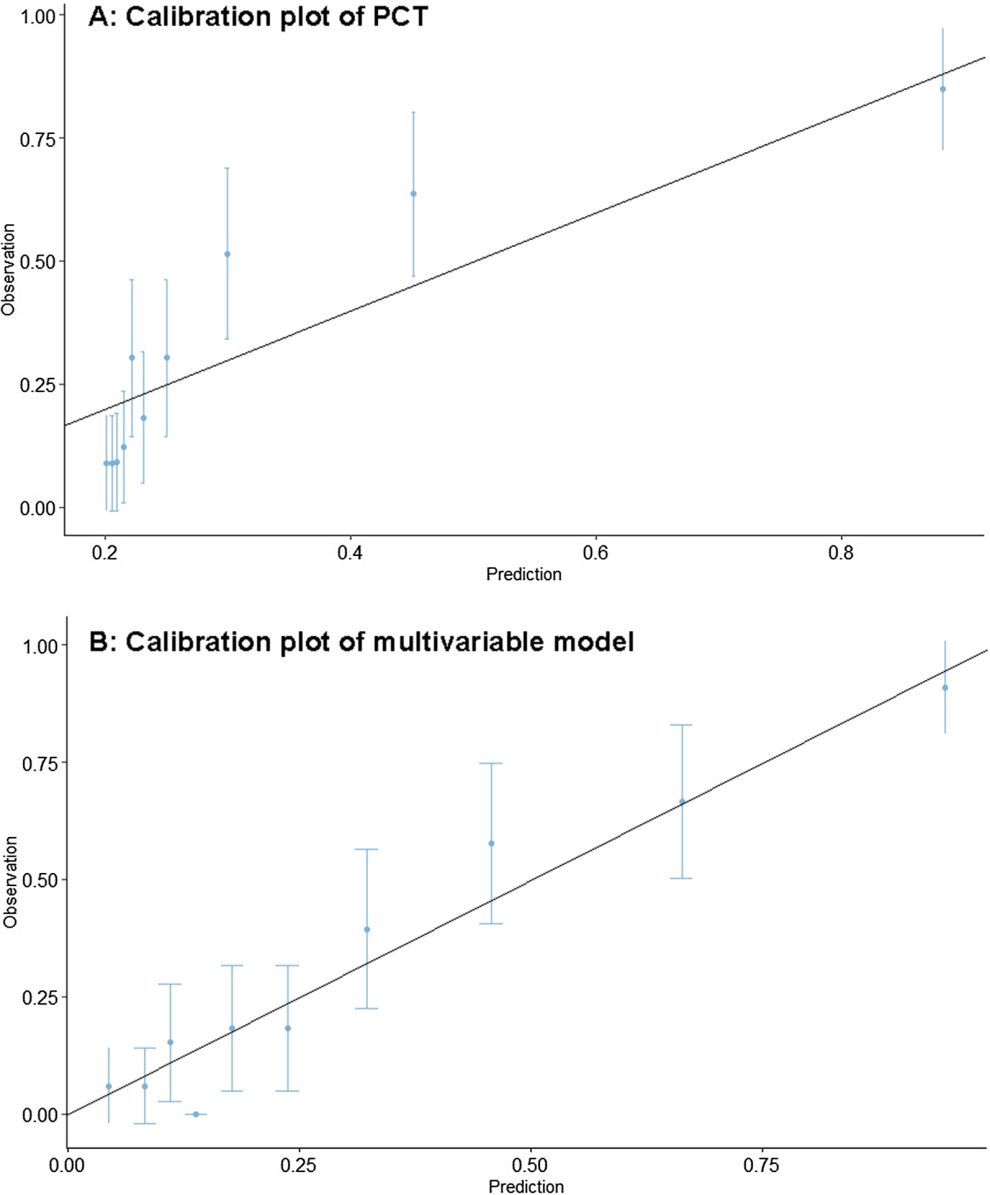
**A** Calibration plot of PCT. **B** Calibration plot of multivariable model. *PCT* Procalcitonin

On the univariate analysis of PCT on a severe COVID-19 infection, the Hosmer-Lemshow test showed a bad fit (p = 0.003) and the calibration plot showed a slope of 1.00 (95% CI: 0.64–1.42) and intercept of 0.00 (95% CI: − 0.42–0.48) (Fig. 2A). The Hosmer-Lewshow test of the multivariable logistic regression model showed a good fit (p = 0.16) and the calibration plot showed a slope of 1.00 (CI: 0.76–1.27) with an intercept of 0.00 (CI: − 0.35–0.36) (Fig. 2B).

For the primary outcome, the OR of PCT at these cutoff points were respectively 9.76 (95% CI: 5.79–16.83), 12.12 (95% CI: 6.61–23.2) and 14.40 (95% CI: 6.45–36.77). The ORs of PCT at the different cut-off points of 0.25 ng/mL, 0.5 ng/mL and 1.0 ng/mL for the secondary outcomes are shown in Table 3. On hospital admission, the OR of PCT was 14.68 (95% CI: 5.27–61.11) at a cutoff point of 0.25 ng/mL and 22.78 (95% CI: 4.88–406.11) at a cutoff point of 0.5 ng/mL. Since there were no patients who were discharged home with a PCT of higher than 1.0, we could not calculate the OR of PCT at a cutoff of 1.0. For ICU admission, the OR of PCT at the previously mentioned cutoff points were 5.94 (95% CI: 3.36–10.79), 7.7 (95% CI: 4.24–14.2) and 8.93 (95% CI: 4.42–18.51) respectively. For mortality the ORs were 9.72 (95% CI: 5.2–19.08), 9.38 (95% CI: 5.08–17.68) and 7.93 (95% CI: 3.92–16.29) respectively.

**Table 3:** Procalcitonin at different cutoff points on primary and secondary outcomes

Outcome	Cut-off value PCT	Odds ratio	Confidence interval	Sensitivity	Specificity	Negative predictive value	Positive predictive value
Severe outcome	Continuous scale	4.19	(2.52–7.69)				
	0.25 (ng/mL)	9.76	(5.79–16.83)	0.67	0.82	0.84	0.63
	0.5 (ng/mL)	12.12	(6.61–23.2)	0.49	0.92	0.79	0.75
	1 (ng/mL)	14.40	(6.45–36.77)	0.31	0.96	0.75	0.82
Hospital admission	Continuous scale	1667.00	(67.91–98,005.22)				
	0.25 (ng/mL)	14.68	(5.27–61.11)	0.40	0.95	0.28	0.97
	0.5 (ng/mL)	22.78	(4.88–406.11)	0.25	0.98	0.25	0.98
	1 (ng/mL)	NA	NA	0.15	1	0.22	1
ICU admission	Continuous scale	1.15	(1.05–1.3)				
	0.25 (ng/mL)	5.94	(3.36–10.79)	0.66	0.74	0.90	0.39
	0.5 (ng/mL)	7.70	(4.24–14.2)	0.53	0.87	0.88	0.50
	1 (ng/mL)	8.93	(4.42–18.51)	0.36	0.93	0.85	0.6
Mortality	Continuous scale	1.22	(1.08–1.43)				
	0.25 (ng/mL)	9.72	(5.2–19.08)	0.75	0.76	0.93	0.41
	0.5 (ng/mL)	9.38	(5.08–17.68)	0.57	0.87	0.90	0.50
	1 (ng/mL)	7.93	(3.92–16.29)	0.36	0.93	0.86	0.55

*ICU* intensive care unit, *NA* not available

## Discussion

In this study we found that elevated PCT levels are associated with an increase in the combined outcome of ICU admission and mortality. When treating a COVID-19 patient in the ED with an elevated PCT, the treating physician should not only look for possible bacterial coinfections but also be cautious of disease progression. Based on these findings, physicians should beware that patients with elevated PCT levels in the ED may have an increased risk of becoming severely ill. Although these findings need to be validated before PCT levels can be used to guide clinical decision-making on disposition of COVID-19 patients, PCT levels may serve as an early warning sign of possible clinical deterioration. Due to a low sensitivity of PCT on all outcomes, normal PCT levels should not be used in disposition decisions in COVID-19 patients in the ED.

Our findings are supported by other studies, that showed that hospitalized COVID-19 patients with severe infections had higher PCT levels [15–17]. Only a few studies focused on the use of PCT in patients with a COVID-19 infection in an ED setting. Nazerian et al. found that PCT was not useful in diagnosing COVID-19 in the ED. However, the association PCT and of disease severity was not analysed [18]. Surme et al. investigated predictors of ICU admission and mortality and also found that high PCT were associated with an increase of the composite outcome of ICU admission and mortality [19]. Kaal et al. also showed similar results in a cohort of 142 patients, and showed that patients with PCT levels above 0.1 ng/mL have an elevated risk of a severe infection [20].

PCT is primarily used as biomarker for bacterial infections. The findings of this study show a new use for PCT in the specific group of COVID-19 patients as marker of disease severity. Even after correcting for bacterial coinfections and inflammatory markers CRP and d-dimer, elevated PCT levels remained associated with a severe COVID-19 infection. Our finding that PCT is highly elevated in severe COVID-19 infections can be explained when looking at the pathways of synthesis of PCT [10]. PCT synthesis is upregulated by different cytokines such as interleukine-6 and TNF-alpha. Because hyperinflammation is shown to be an important factor in progression of COVID-19 infections, the dysregulated immune response may also trigger PCT production [4]. Similar results of elevated PCT levels were found in patients with isolated severe influenza virus infections, findings that support the hypothesis that PCT is a marker of hyperinflammation [21].

At different cut-off values, PCT levels showed a high specificity for the hospital admission, ICU admission and mortality. On hospital admission, the lowest cut-off value of 0.25 ng/mL resulted in a specificity of 95%. This finding could be used as an argument to use PCT as support tool for hospital disposition decisions, because the majority of patients with a PCT above 0.25 ng/mL were admitted to the hospital. The sensitivity of PCT on all outcomes was low. Therefore, PCT is less suitable for being incorporated it in a rule out decision support tool of severe COVID-19 infections in the ED. Different cut-off values of PCT are recommended in distinguishing bacterial from viral infections, also ranging from 0.25 to 1.0 ng/mL [14]. The OR of PCT on hospital admission was 1667 (95% CI: 68–98,005). This high OR can be explained by the exponential increase in PCT levels in patients with severe infections. In our cohort, only 67 (20%) patients were discharged from the ED and the highest PCT level measured in these patients was 0.82 mg/mL. The patients that were admitted to the hospital also included the group that required ICU admission or died and several patients had a PCT levels above 10 ng/mL. This large difference in PCT levels between these groups resulted in the high OR.

### Limitations

A limitation of this study is its retrospective design. Patients were included if PCT was measured in the ED. PCT was only measured in patients where the primary reason of the ED visit was suspected COVID-19 infection. Patients that were initially not suspected of having COVID-19, but were diagnosed with a COVID-19 infection nonetheless, were not included in this study.

In the secondary outcomes, the subgroups have a relatively small size, which influences the generalizability of the findings.

The patient population consisted solely of confirmed COVID-19 patients. Therefore, the results are not biased by patients who visited the ED with a suspected COVID-19 infection but were diagnosed with an alternative diagnosis. However, the results of this study cannot be used for distinguishing a COVID-19 infection from other viral or bacterial infections [18].

This study was performed in a cohort of patients who visited the ED in 2020, when no vaccinations against SARS-CoV-2 were available. It is unclear if these results are generalizable to the current ED setting, where the majority of patients is vaccinated [22].These results require validation in vaccinated patients.

Using only PCT as single predictor of the primary outcome showed a poor goodness-of-fit and the results may not be valid in other cohorts. Therefore, these results have to be validated in other cohorts. Because PCT has a skewed distribution of < 0.50 ng/mL in non-severe patients and from 0.50 up to 58 ng/mL in severely infected patients, the results may be biased by the patients with high PCT values. This can be corrected by using the natural logarithm of PCT, thereby reducing the range of PCT. The results were similar, but the goodness-of-fit was high (p = 0.91) (Additional file 3).

PCT was prospectively measured in the ED and available to the treating physician, along with other routinely measured laboratory tests. PCT levels may have been used in clinical decision making and influenced disposition decisions.

## Conclusion

High PCT levels are associated with high rates of severe COVID-19 infections in patients with a COVID-19 infection in the ED. The routine measurement of PCT in patients with a COVID-19 infection in the ED may assist physicians in the clinical decision-making process regarding ICU disposition. These results should be validated in a prospective multicenter cohort.

## Supplementary Information

**Additional file 1.** STROBE checklist (Strengthening the reporting of observational studies in epidemiology).

**Additional file 2.** Amount of missing data.

**Additional file 3.** Univariate analysis of natural logarithm of procalcitonin on a severe COVID-19 infection.

**Additional file 4.** Multivariable logistic regression model with different cut-off values of procalcitonin.

## Data Availability

The datasets used and/or analyzed during the current study are available from the corresponding author on reasonable request.

## Abbreviations

COVID-19: Coronavirus disease
SARS-CoV-2: Severe acute respiratory syndrome coronavirus 2
ICU: Intensive care unit
ARDS: Acute respiratory distress syndrome
ED: Emergency department
PCT: Procalcitonin
TNF-alpha: Tumor necrosis factor alpha
RDW: Red cell distribution width
ALAT: Alanine aminotransferase
ASAT: Aspartate aminotransferase
CRP: C-reactive protein
OR: Odds ratio
SD: Standard deviation
IQR: Interquartile range
AUC: Area under the curve
