# Supplementary material for: High procalcitonin levels associated with increased intensive care unit admission and mortality in patients with a COVID-19 infection in the emergency department
Source: BMC Infect Dis. 2022 Feb 21;22:165. doi: 10.1186/s12879-022-07144-5 (PMC8860271; doi:10.1186/s12879-022-07144-5)
Supplement: Supplementary file 1 — Additional file 1. STROBE checklist (Strengthening the reporting of observational studies in epidemiology). [file 12879_2022_7144_MOESM1_ESM.docx]

**Additional file 1**

STROBE checklist (Strengthening the reporting of observational studies in epidemiology).

|  | | Item No. | Recommendation | Page  No. |  |
| --- | --- | --- | --- | --- | --- |
| **Title and abstract** | | 1 | (*a*) Indicate the study’s design with a commonly used term in the title or the abstract | 1, 3-4 |  |
|  |  |  | (*b*) Provide in the abstract an informative and balanced summary of what was done and what was found | 3-4 |  |
| Introduction | | | | |  |
| Background/rationale | | 2 | Explain the scientific background and rationale for the investigation being reported | 5 |  |
| Objectives | | 3 | State specific objectives, including any prespecified hypotheses | 6 |  |
| Methods | | | | |  |
| Study design | | 4 | Present key elements of study design early in the paper | 6 |  |
| Setting | | 5 | Describe the setting, locations, and relevant dates, including periods of recruitment, exposure, follow-up, and data collection | 6 |  |
| Participants | | 6 | (*a*) *Cohort study*—Give the eligibility criteria, and the sources and methods of selection of participants. Describe methods of follow-up | 6-7 |  |
|  |  |  |  |  |  |
| Variables | | 7 | Clearly define all outcomes, exposures, predictors, potential confounders, and effect modifiers. Give diagnostic criteria, if applicable | 7 |  |
| Data sources/ measurement | | 8 | For each variable of interest, give sources of data and details of methods of assessment (measurement). Describe comparability of assessment methods if there is more than one group | *7* |  |
| Bias | | 9 | Describe any efforts to address potential sources of bias | NA |  |
| Study size | | 10 | Explain how the study size was arrived at | 6 |  |
| Quantitative variables | | 11 | Explain how quantitative variables were handled in the analyses. If applicable, describe which groupings were chosen and why | 8 |  |
| Statistical methods | | 12 | (*a*) Describe all statistical methods, including those used to control for confounding | 8 |  |
|  |  |  | (*b*) Describe any methods used to examine subgroups and interactions | 8 |  |
|  |  |  | (*c*) Explain how missing data were addressed | 8 |  |
|  |  |  | (*d*) *Cohort study*—If applicable, explain how loss to follow-up was addressed | NA |  |
|  |  |  | (*e*) Describe any sensitivity analyses | 8 |  |
| Participants | | 13 | (a) Report numbers of individuals at each stage of study—eg numbers potentially eligible, examined for eligibility, confirmed eligible, included in the study, completing follow-up, and analysed | 9 |  |
|  |  |  | (b) Give reasons for non-participation at each stage | 9 |  |
|  |  |  | (c) Consider use of a flow diagram | Figure 1 |  |
| Descriptive data | | 14 | (a) Give characteristics of study participants (eg demographic, clinical, social) and information on exposures and potential confounders | 9 |  |
|  |  |  | (b) Indicate number of participants with missing data for each variable of interest | 9 |  |
|  |  |  | (c) *Cohort study*—Summarise follow-up time (eg, average and total amount) | NA |  |
| Outcome data | | 15 | *Cohort study*—Report numbers of outcome events or summary measures over time | *9* |  |
|  |  |  |  |  |  |
|  |  |  |  |  |  |
| Main results | | 16 | (*a*) Give unadjusted estimates and, if applicable, confounder-adjusted estimates and their precision (eg, 95% confidence interval). Make clear which confounders were adjusted for and why they were included | 9-10 |  |
|  |  |  | (*b*) Report category boundaries when continuous variables were categorized | 10 |  |
|  |  |  | (*c*) If relevant, consider translating estimates of relative risk into absolute risk for a meaningful time period | NA |  |
| Other analyses | 17 | Report other analyses done—eg analyses of subgroups and interactions, and sensitivity analyses | | 10 | |
| Discussion | | | | | |
| Key results | 18 | Summarise key results with reference to study objectives | | 11 | |
| Limitations | 19 | Discuss limitations of the study, taking into account sources of potential bias or imprecision. Discuss both direction and magnitude of any potential bias | | 12-13 | |
| Interpretation | 20 | Give a cautious overall interpretation of results considering objectives, limitations, multiplicity of analyses, results from similar studies, and other relevant evidence | | 11-12 | |
| Generalisability | 21 | Discuss the generalisability (external validity) of the study results | | 11-13 | |
| Other information | |  | | | |
| Funding | 22 | Give the source of funding and the role of the funders for the present study and, if applicable, for the original study on which the present article is based | | 15 | |
