# Supplementary material for: High procalcitonin levels associated with increased intensive care unit admission and mortality in patients with a COVID-19 infection in the emergency department
Source: BMC Infect Dis. 2022 Feb 21;22:165. doi: 10.1186/s12879-022-07144-5 (PMC8860271; doi:10.1186/s12879-022-07144-5)
Supplement: Supplementary file 2 — Additional file 2. Amount of missing data. [file 12879_2022_7144_MOESM2_ESM.docx]

**Additional file 2**

| **Variable** | **Amount missing** | **Percentage missing** |
| --- | --- | --- |
| Admission length | 1 | <1 |
| ASAT | 3 | 1 |
| ALAT | 8 | 2 |
| Total bilirubine | 5 | 2 |
| Thrombocytes | 1 | <1 |
| Lactate | 19 | 6 |
| Temperature | 22 | 7 |
| Heartrate | 13 | 4 |
| Respiratory rate | 57 | 17 |
| SpO2 | 14 | 4 |
| Blood pressure | 19 | 6 |
|  |  |  |
|  | | |

**Additional table 1:** Amount of missing data.

ASAT: Aspartate aminotransferase. ALAT: Alanine-aminotransferase.
