# Supplementary material for: High procalcitonin levels associated with increased intensive care unit admission and mortality in patients with a COVID-19 infection in the emergency department
Source: BMC Infect Dis. 2022 Feb 21;22:165. doi: 10.1186/s12879-022-07144-5 (PMC8860271; doi:10.1186/s12879-022-07144-5)
Supplement: Supplementary file 3 — Additional file 3. Univariate analysis of natural logarithm of procalcitonin on a severe COVID-19 infection. [file 12879_2022_7144_MOESM3_ESM.docx]

**Additional file 3**

Univariate analysis of procalcitonin on a severe COVID-19 infection. Odds ratio with 95% confidence interval of the natural logarithm of procalcitonin: 2.79 (2.20 – 3.63).

The Hosmer-Lemeshow test showed a high goodness-of-fit: p=0.91


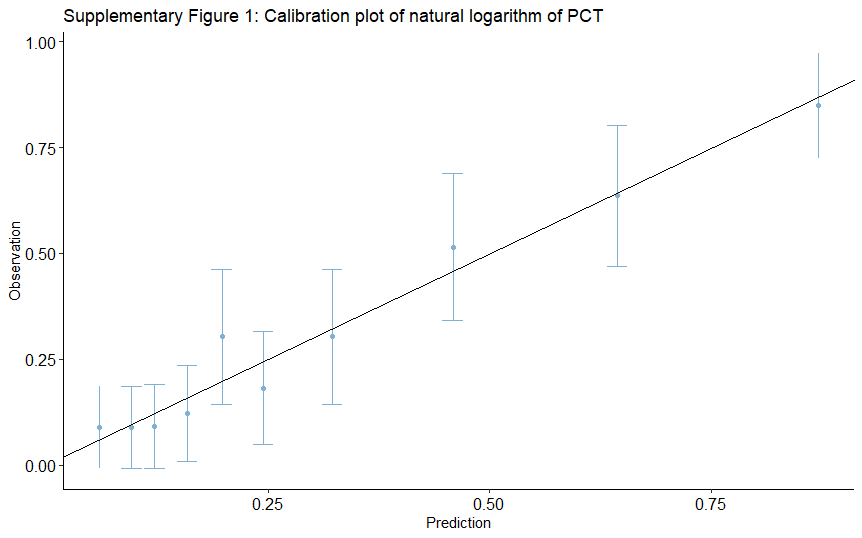


**Figure S1:** Calibration plot of the natural logarithm of procalcitonin. Slope: 1.00 (0.77 – 1.26), intercept: 0.00 (-0.32 – 0.34).

PCT: Procalcitonin
