# Supplementary material for: High procalcitonin levels associated with increased intensive care unit admission and mortality in patients with a COVID-19 infection in the emergency department
Source: BMC Infect Dis. 2022 Feb 21;22:165. doi: 10.1186/s12879-022-07144-5 (PMC8860271; doi:10.1186/s12879-022-07144-5)
Supplement: Supplementary file 4 — Additional file 4. Multivariable logistic regression model with different cut-off values of procalcitonin. [file 12879_2022_7144_MOESM4_ESM.docx]

**Additional file 4**

| Predictor | Odds Ratio | Confidence interval |
| --- | --- | --- |
|  |  | |
| Procalcitonin at different cut-off values |  |  |
| <0.25 ng/mL | Reference | Reference |
| 0.25 – 0.50 ng/mL | 2.52 | 1.07 – 5.85 |
| 0.50 – 1.00 ng/mL | 3.58 | 1.33 – 9.78 |
| >1.00 ng/mL | 9.56 | 3.56 – 28.09 |
| Bacterial coinfection | 1.95 | 0.78 - 4.77 |
| Sex: male | 1.31 | 0.70 - 2.48 |
| Age | 1.02 | 1.00 - 1.05 |
| Comorbidity: any | 0.93 | 0.42 - 2.13 |
| CRP | 1.00 | 1.00 - 1.01 |
| D-dimer | 1.24 | 1.12 - 1.41 |

**Table S2:** multivariable logistic regression model with different cut-off values of procalcitonin

CRP: C-reactive protein.
